# Re-Evaluation of the Ultrastructural Localization of Tonic GABA-A Receptors

**DOI:** 10.3390/ph19010025

**Published:** 2025-12-22

**Authors:** Abraham Rosas-Arellano

**Affiliations:** Imaging Unit, Institute of Cellular Physiology, National Autonomous University of Mexico, Mexico City 04510, Mexico; arosasar@ifc.unam.mx

**Keywords:** extrasynaptic, perisynaptic, tonic inhibition, tonic conductance, tonic activity

## Abstract

Cell membrane receptors play key roles in physiological and pathological processes, yet the mechanisms governing their expression and distribution across the plasma membrane remain not completely understood. Broadly, membrane receptors can be categorized into phasic and tonic receptors. Tonic GABA-A receptors have attracted considerable interest due to their distinct molecular composition and their capacity to mediate highly sensitive, sustained inhibitory responses in the presence of ambient GABA. Traditionally, these receptors have been described as residing in peri- and extrasynaptic regions, where they are thought to sense GABA spillover and generate tonic inhibition. However, evidence accumulated over several decades has challenged this canonical view. Multiple studies have reported activity-dependent and pathology-associated relocalization of tonic GABA-A receptor subunits from their typical peri- and extrasynaptic domains into the synaptic cleft. This phenomenon has been documented in both in vivo and in vitro models, yet major questions remain regarding its occurrence, underlying mechanisms, functional significance, and adaptive value. This review synthesizes current evidence and highlights ongoing controversies surrounding the ultrastructural localization of tonic GABA-A receptors. Based on an exhaustive search of the PubMed database, this review summarizes key findings from studies investigating the subcellular distribution of these receptors and discusses emerging perspectives on their potential synaptic presence.

## 1. Introduction

### 1.1. Tonic Activity

The nervous system involves a series of physiological events of both excitatory and inhibitory nature. Both are crucial for the communication of neuronal circuits and the maintenance of intracellular and extracellular balance. The activation of both pathways depends primarily on a specific stimulus and generates responses with a wide range of time intervals [1].

In the intricate functional neuronal network, two types of action potentials with differential states of activation and deactivation are generally described: (a) Action potentials that present high frequency and short-duration bursts, showing a high-amplitude electrophysiological pattern that disappears quickly. These physiological events have been called phasic activity [2,3]; (b) Action Potentials that exhibit low-frequency and sustained activity, which have been assigned the term tonic activity [2,4].

Electrophysiological experiments performed in *Xenopus laevis* oocytes microinjected with bovine retinal RNA showed that, within a 4 min period, γ-aminobutyric acid (GABA)-dependent currents with slow desensitization (approximately 12%) and low amplitudes were generated compared to those obtained in oocytes injected with RNA from the cerebral cortex; the retina has been recognized for its substantial expression of tonic GABA receptors, whereas the cortex seems to be more abundant in phasic resources. For GABA phasic currents, approximately 92% desensitization has been reported, and both phasic and tonic currents have been recorded in the presence of GABA [5]. Tonic currents, sometimes also called tonic conductance or tonic activity, are dependent on a wide variety of neuronal stimuli, including (1) voltage changes, (2) intracellular signaling, and (3) the activity of ligand-binding receptors, such as glutamate, dopamine, and GABA, just to name a few [6,7]. As expected, tonic activity does not exclusively result in inhibitory activity, and it is also found as an excitatory resource in, for example, dopamine and NMDA pathways, where it has been shown that phasic activity requires micromolar concentrations while tonic activity requires nanomolar concentrations of NMDA [1,8].

Tonic activity is present in virtually all structures of the nervous system, influencing neuronal firing patterns and modulating synaptic transmission. Molecularly, it has been associated with the functional participation of metabotropic and ionotropic receptors [9]. The former are G protein-coupled transmembrane receptors; their activation triggers the involvement of a series of second messengers, such as cAMP and IP3, which are molecules that, in turn, modulate the activity of membrane channels to allow the flow of ions [10]. Tonic ionotropic receptors, which are the other source of tonic activity, are channel-anchored proteins of plasma membranes. Unlike metabotropic receptors, they respond more quickly because they do not depend on a signaling cascade, but, like the latter, their activity can manifest itself through long-lasting firing behaviors [4,11].

### 1.2. Subcellular Distribution of Tonic Resources

**Figure 1:**
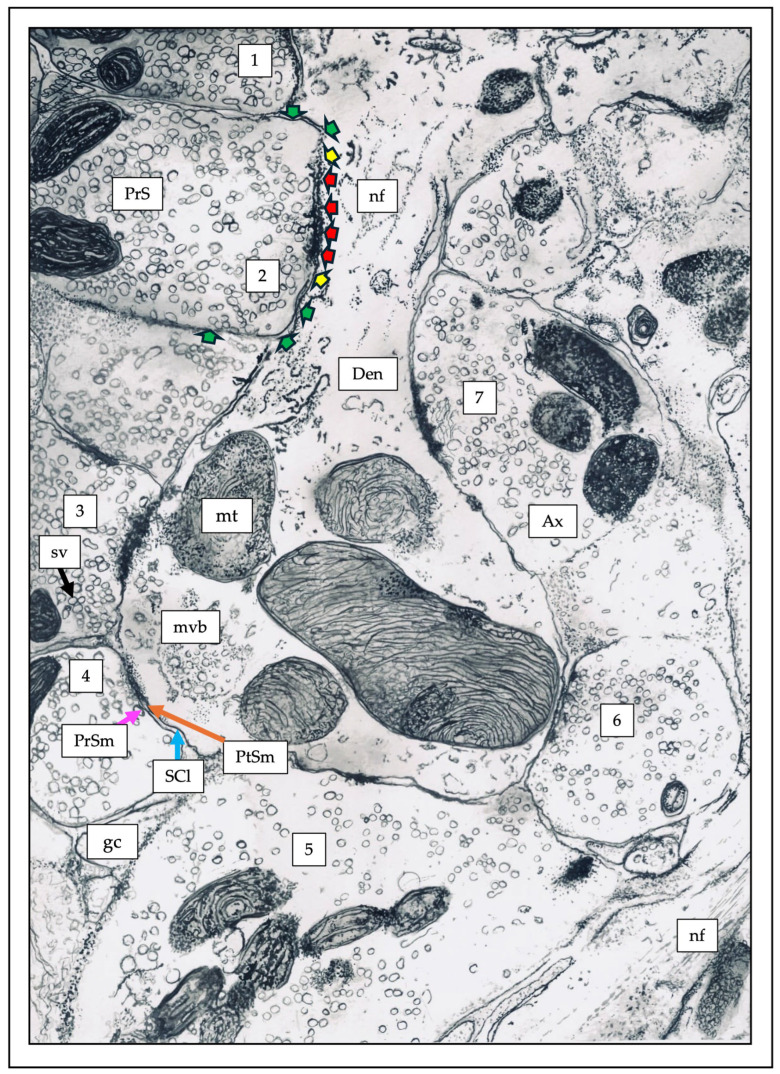
The synapse. This is a Chinese ink painting of an axodendritic synapse, based on an electron micrograph from Peters’ book [12]. It shows a dendritic terminal connected to at least to seven axons, or terminal boutons, where the following can be distinguished: (1) synaptic vesicles (sv), into the presynaptic terminals (PrS); (2) presynaptic membrane (PrSm, magenta arrow); (3) synaptic cleft (SCl, blue arrow), the space between the presynaptic and postsynaptic membranes; (3) postsynaptic membrane (PtSm, orange arrow), and (4) electron density (red arrows), which reveals the area of the active zone. The areas shown by the yellow arrows are called the perisynapse, and the area indicated by the green arrows is called the extrasynapse. These three locations, synaptic, perisynaptic, and estrasynaptic, are not exclusive to the postynaptic membrane; they are also areas of the presynaptic region. Other abbreviations: gc (glial cell), mt (mitochondria), mvb (multi vesicular bodies), nf (neurofilaments), Den (dentrite), Ax (axon).

Another feature appears to be the subcellular location of the receptors involved in tonic activity. Neuronal communication occurs primarily at the synaptic junction, a very well-defined region consisting of clearly distinguishable zones, as shown in the painting of an electron micrograph in Figure 1 (based on [12]). At the subcellular level, by convention, all molecules or activities those found in the active zone are called synaptic, and those flanking the active zone are assigned the term perisynaptic, while the others that are outside, near or far, of the synaptic junction and perisynaptic sites are called extrasynaptic; therefore, the areas corresponding to the synaptic region will always be smaller compared to those covering the peri- and extrasynaptic regions, the latter being the one that covers the largest area [11,13,14]. Accordingly, neuronal receptors are classified based on the synaptic nomenclature, i.e., (a) synaptic, (b) perisynaptic, and (c) extrasynaptic, depending on their physical or functional location at the time of observation. Some immunogold studies have consistently reported extrasynaptic and perisynaptic tonic receptors such as glutamate, potassium, and GABA, although they have also been reported in synaptic regions, apparently at a low abundance [15,16,17,18].

### 1.3. γ-Aminobutyric Acid

GABA has played an important role in the life of a large number of species throughout evolution; it has been reported in bacteria, fungi, plants, invertebrates, and vertebrates. This amino acid is present in virtually all life forms, and the function and structure of its receptors are highly conserved throughout vertebrate evolution, indicating that its role in the functioning of the nervous system is irreplaceable [19,20,21]. The function of GABA is primarily inhibitory and is present in all stages of vertebrate life; however, GABA exhibits excitatory activity in response to certain factors, such as age, health, or disease states [11,22,23]. In this sense, the homopentameric cation-selective receptor of the cys-loop family ligand-gated channel 35 is exclusively activated by GABA in *Caenorhabditis elegans* [24]; whereas neonatal hippocampal neurons show GABA-dependent depolarizations rather than hyperpolarization due to a high chloride concentration within the cell [23]. Reports of GABA as a biomolecule in the nervous system date back to 1950, when Roberts and his colleague first described it as part of a population of free amino acids in the brain [25]. Subsequent studies indicate that its activity corresponds to that of a chemical messenger involved in inhibitory neuronal communication [26,27,28]. GABA is a non-protein amino acid that is synthesized from another amino acid, glutamate, which in turn is the most abundant excitatory neurotransmitter in the brain. Almost 40% of all neurons are glutaminergic neurons, and more than 90% of neurons have glutamate receptors, while around 15% of all neurons are GABAergic neurons, and 60–75% of neurons translate into GABA receptors, the most widespread inhibitory receptors in the central nervous system [3,25,29].

GABA synthesis occurs through the enzyme glutamate decarboxylase, known as GAD, and the sole presence of this enzyme determines the GABAergic identity of a cell. GAD is present in two isoforms of different molecular weights, i.e., 65 and 67 kDa, which are called GAD65 and GAD67, respectively [30,31,32]. GABA is also synthetsized by monoamine oxidase B in glial cells, and these cells also express, and these cells in turn express GABA receptors as well [33,34]. Although it had traditionally been postulated that neurotransmitters were differentially released by a single neuronal identity, the co-release of glutamate–GABA in the same synaptic vesicle is currently recognized [35]. Functionally, as mentioned before, GABA plays an important role as a primary inhibitory signal; its participation contributes to maintaining neuronal on–off balance through two general types of receptors: GABA-A, which forms pentameric channels in plasma membranes, and others designated as GABA-B, which forms metabotropic receptors composed by two subunits called GABABR1 and GABABR2; these metabotropic GABA receptors are couple with ion channels via guanine nucleotide-binding proteins and second messengers [3,36,37]; GABA-A receptors are described in the next section.

### 1.4. γ-Aminobutyric Acid Sub-Type a Receptors

**Figure 2:**
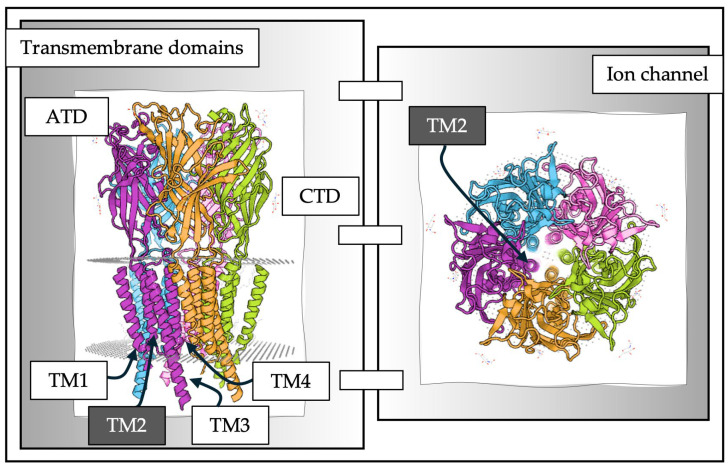
A GABA-A receptor. GABA-A receptors are lipid-anchored membrane proteins. They possess a large extracellular N-terminal domain (ATD) of 220–250 residues, which houses the agonist-binding site. They also possess four transmembrane domains called TM1, TM2, TM3, and TM4 (the transmembrane domains are indicated only for the purple subunit) and a short extracellular C-terminal domain (CTD) (left). The five subunits (each one represented with different colors) of ionotropic GABA receptors present their TM2 domain on the inner surface of the pentamer. The pentameric assembly allows for the formation of a circular area in the form of a dynamic pore that, upon binding to GABA or other agonist, allows conformational changes in key amino acids of TM2 to allow ion permeation (right), according to Rossokhin and colleagues, the 2′ (desensitization residue), 9′ (activation gate) and 20′ amino residues appear to control the pore diameter [2]. These models were developed using the Swiss-model using a template of the human GABA-A rho1 subunit obtained using cryo-electron microscopy. SWISS-MODEL Repository, Switzerland, https://swissmodel.expasy.org/repository?start=0&rows=50&query=gabrr1 (accessed on 4 August 2025).

Classified as GABA-A receptors are a family of channel proteins that are made up of five subunits, each of which comprises four transmembrane domains; one of these four domains forms, together with its homologs of the remaining four subunits, a pore for the chloride ion (Figure 2). Based on their structure, they have been included in the ligand-gated ion channel of the Cys-loop superfamily that includes other ion channels such as nicotinic acetylcholine, glycine, serotonin, and zinc-activated receptors [38,39]. GABA-A receptors are widely distributed throughout the brain, brainstem, and spinal cord, primarily in postsynaptic membranes, but there is also evidence that they are located at presynaptic and extrasynaptic sites [40,41].

The subunits that make up the pentamer can include any of the following nineteen subunits: six α, three β, three γ, one δ, three ρ, one ε, one π, and one θ. GABA-A ρ receptors are sometimes referred to as GABA-C receptors due to their distinctive pharmacological profile [42,43]. However, NC-IUPHAR classifies ρ subunits as GABA-A based on structural and functional criteria [44]. The most widespread GABA-A subunits are α1, whereas the less widespread ones are ε, π, and θ. The α1 is common in the interneurons of cerebral cortex, basal ganglia, and hippocampus, as well as in the GABAergic projection neurons of basal ganglia; the ε subunit was detected in the rat brain and heart, while the θ subunit was detected in the mouse brain, lung, and spleen, and both subunits are found in the locus ceruleus of postnatal rats [41,45]. Finally, the π subunit is expressed in reproductive tissue, the pancreas, and in certain cancers as pancreatic adenocarcinoma [42,44,46]. Considering the 19 subunits that compose it, the GABA-A is composed of homopentameric or heteropentameric subunits, even within the same cell, with the heteropentameric form being the most common configuration. A GABA-A pentamer typically weighs between 260 and 295 kDa, meaning that each subunit weighs between 52 and 59 kDa, and the best-known stoichiometries, representing approximately 60%, have been described as 2α (α1), 2β (β2), and 1γ (γ2); the latter is usually replaced by the ε or δ subunit, whereas the ρ pentamers, despite being widely distributed in the nervous system, constitute a small population of GABAergic pentamers and are known to form homo- or heteropentamers [2,7,47]. The diversity of GABA-A receptors is such that, in deletion studies of their subunits that compose them, it seems that other subunits compensate for the loss, causing poorly developed pathologies or the absence of an apparent phenotype in experimental models.

It is evident that GABA-A receptors are highly heterogeneous, and it is believed that this diversity can lead to the formation of more than 500 varieties of pentamers and, therefore, generate a wide range of electrical responses. Furthermore, alternative variants of at least nine subunits have been reported [48,49]. These receptors are also traditionally located at different synaptic sites (synapse, perisynapse, and extrasynapse); both the presynapse and the postsynapse, a wide range of functional responses [11,50,51]. This great diversity of response is what puzzles anyone who tries to study them. Moreover, this GABAergic diversity–i.e., homo- or heteropentameric, from presynaptic to postsynaptic, and from phasic to tonic–could explain how, despite excitatory resources being more abundant in non-pathological states, GABA manages to maintain a balance between excitation and inhibition despite not showing the same abundance as its counterpart glutamate.

### 1.5. Molecular Identity of GABA-A Tonic Subunits

Tonic GABA-A pentamers form homoreceptors or heteroreceptors, with the latter either including or excluding phasic subunits; however, their stoichiometry is determined by the inclusion of one or more of the following subunits: α4–6, β3, δ, and ρ1–3. As mentioned above, it is noteworthy that tonic GABA-A receptors have been described to be differentially positioned in peri- and extrasynaptic regions: (1) they are located flanking the synaptic cleft, either on one or both sides, or (2) they are distributed close to or far from the well-defined synaptic area [11,52]. The tonic activity of GABA-A, as expected, shows differential characteristics based on the ligand sensitivity and the stoichiometry of the pentameric receptor involved. Thus, there is a wide variety of temporal ranges of responses within the tonic activity of GABA-A due to the diversity of subunits that make up the pentamer, which result in the pharmacological and functional differences between them. For example, it is well known that L-655,708, α5IA, Ro15-4513, MRK-016, RO4938581, and RY-80, the negative allosteric modulators, are selective for the α5 subunit [2,53]. On the other hand, depending on their stoichiometry, some tonic GABA receptors depend on micromolar GABA concentrations for their activation; thus, their EC50 can vary from a few micromoles to hundreds of micromoles. Some tonic GABAergic receptors stand out as a very low concentration of GABA is required to unblock their channels, they activate the ionic flow more slowly, and they exhibit a slower desensitization than other GABA-A tonic receptors, as observed in GABA-A ρ1 homomers [54]. Although the diversity of stimuli is evident, the tonic response is always persistent in the presence of an agonist, and slow desensitization occurs until its deactivation, thus contributing to the long-term modulation of neuronal excitability, which is essential in a mature nervous system for the synaptic inhibition and synchronization of neuronal networks [2,5].

**Figure 3:**
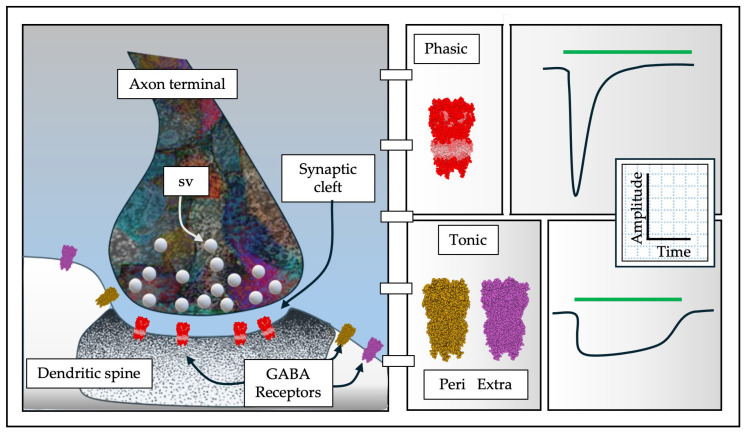
GABA-A phasic vs. GABA-A tonic. The GABA-AR phasic pentamers are found in the active zone of the synapse (pentamers in red), while the tonic subunits are found in the perisynaptic (golden pentamer) and extrasynaptic (magenta pentamer away from the synaptic region) (left). From these positions, both phasic and tonic show distinctive electrophysiological activities, while phasic receptors show high amplitude currents with short response periods, tonic receptors show lower amplitude currents but persist in the presence of the agonist (green line) (right).

The tonic GABA-A subunits have increasingly garnered the interest of research groups in recent years, and these subunits fulfill the classic characteristics of tonic receptors (Figure 3). Tonic GABA-AR have been described in various areas of the central nervous system, both during its development and in adulthood [7,55]. Interestingly, fluctuations in tonic subunits’ expression have been reported throughout different life stages [56,57,58].

### 1.6. Summary of the Characteristics of Each GABA-A Tonic Subunit

#### 1.6.1. GABA-A α4

The subunit called GABRA4 in humans and Gabra4 in rodents consists of 554 and 552 amino acids, respectively, and it has a molecular weight of approximately 60 kDa. It has been described to contribute to tonic activity in various brain areas, including the dentate gyrus, thalamus, striatum, cerebral cortex, CA1, nucleus accumbens, olfactory tubercle, superior colliculus, brainstem, and olfactory bulb. The formation of pentamers with the subunits has been described as follows: β1,γ2; β1,δ; β2γ2; β2,δ; β3,γ2; and β3,δ. Pathologically, it is associated with autism spectrum disorders and alcohol consumption [2,59,60,61,62].

#### 1.6.2. GABA-A α5

The subunits called GABRA5 in humans and Gabra5 in rodents consist of 462 amino acids in humans and 463 and 464 amino acids in mice and rats, respectively. The molecular weight of this subunit is typically around 55 kDa. GABRA5 has been widely present in peri- and extrasynaptic regions through several brain areas such as the olfactory system, neocortex, subiculum, dentate gyrus, CA1, CA3, amygdala, striatum, thalamus, hypothalamus, and brainstem. The receptors containing the α5 subunit generally consist of 2α, 2β, and 1γ subunits and have been reported to form pentamers together with β1,γ2; β2,γ2; β2,γ3; and β3;γ2 subunits. Its pathophysiological relevance is associated with autism spectrum disorder, bipolar disorders, and Huntington’s disease [2,11,17,62,63].

#### 1.6.3. GABA-A α6

The subunit called GABRA6 in humans and Gabra6 in rodents consists of 453 amino acids in both humans and rodents, and the α6 subunit has a molecular weight of approximately 51 kDa. This subunit has been described in synaptic and extrasynaptic regions of the cerebellum, the cochlear nucleus, and mossy fibers, where it forms pentamers with the subunits β1,γ2; β2,γ1; β2,γ2; β2,δ; β3γ2; and β3,δ. Its pathological relevance is unclear [2,62,64].

#### 1.6.4. GABA-A β3

The subunit called GABRB3 in humans and Gabrb3 in rodents consists of 473 amino acids, both in humans and rodents. Its molecular weight is usually between 53 and 58 kDa. The β3 subunit has been widely described in brain areas such as the cortex, hippocampus, basal ganglia, olfactory bulb, hypothalamus, cerebellum, brain stem, and spinal cord. The β3 subunit has been reported to form pentamers together with the subunits α1,γ1; α1,γ2; α1,δ; α2,γ2; α3,γ2; α4,γ2; α4,δ; α5,γ2; α6,γ2; and α6,δ. Its pathophysiological relevance is associated with Alzheimer’s disease, autism spectrum disorder, Angelman syndrome, Huntington’s disease, insomnia, and epilepsy [2,11,17,62,65,66].

#### 1.6.5. GABA-A δ

The subunit called GABRD in humans and Gabrd in rodents consists of 452 amino acids in humans and 449 in rodents. The δ subunit is a protein with a molecular weight of around 50 kDa. This subunit has been described principally as an extrasynaptic subunit of many regions of the brain, such as the hippocampus, cerebellum, thalamus, striatum, cortex, olfactory bulb, cochlear nucleus, and mossy fibers. The δ subunit has been reported to form pentamers with the subunits α1,β2; α1;β3; α4,β1; α4,β2; α4,β3; α6,b2; and α6,β3. Its pathophysiological relevance is associated with Alzheimer’s disease, Huntington’s disease, and epilepsy [2,11,17,62,66,67].

#### 1.6.6. GABA-A ρ1

The ρ1 subunit, called GABRR1 in humans and Gabrr1 in rodents, consists of 479 amino acids in humans and 480 in rodents, with a molecular weight of around 50 kDa. It has been described mainly as an extrasynaptic subunit in neurons as a receptor for glial cells in some regions of the nervous system, such as the hippocampus, cerebellum, striatum, amygdala, cortex, olfactory bulb, retina, corpus callosum, brainstem, and spinal cord. The ρ1 subunit has been reported to form homopentamers and heteropentamers with the subunits α1 and γ2 and the other ρ subunits such as ρ2. Its pathological association is unclear, but some studies suggest its participation in Alzheimer’s disease [7,40,47,62,66,68,69,70].

#### 1.6.7. GABA-A ρ2

The ρ2 subunit, called GABRR2 in humans and Gabrr2 in rodents, consists of 465 amino acids in both humans and rodents. Its molecular weight is usually around 51 kDa; however, some sources also cite a molecular weight of 54 kDa. This subunit has been described principally as an extrasynaptic subunit in some regions of the nervous system, such as the neurons and glial cells of the hippocampus, cerebellum, striatum, retina, corpus callosum, amygdala, cortex, brainstem, and spinal cord. The ρ2 subunit has been reported to form homopentamers, and its heteropentameric form is unreported, although the ρ2 subunit has been reported to be associated with subunit ρ1. Its pathophysiological relevance is associated with Huntington’s disease [7,11,17,40,62,70,71].

#### 1.6.8. GABA-A ρ3

The ρ3 subunit, called GABRR3 in human genes and Gabrr in rodent genes, consists of 467 amino acids in humans and 464 in rodents, and its molecular weight is usually around 51 kDa. Data on this subunit are scarce; it has been described as an extrasynaptic subunit in neurons such as retinal neurons, as well as in the striatum, hippocampus, cerebellum, thalamus, and other structures of the basal ganglia. The ρ3 subunit has been described to form homopentamers, and its heteropentameric form is unreported. Its pathophysiological relevance is associated with Huntington’s disease [7,11,17,62,70,72,73].

## 2. Discussion

### 2.1. Are Tonic GABA-ARs Homogeneously Compartmentalized in the Cell Membrane?

GABA-A tonic receptors are typically located in both the presynaptic and postsynaptic regions; presynaptic receptors are involved in depolarizing of hippocampal mossy fibers terminals, increasing the probability of synaptic release [74], or they are involved in presynaptic inhibition; however, some reports indicate that these receptors facilitate the release of GABA, norepinephrine, adenosine, and luteinizing hormone in central and peripheral tissues [75]. Tonic presynaptic or postsynaptic inhibition is a neurotransmitter pathway-dependent activity and appears to be mediated from peri- and extrasynaptic regions; however, there is a controversy over whether its position is exclusively peri- and extrasynaptic, since there is evidence that some subunits can be found within the active zone of the synapse [17,53,76,77]. This would imply that the subunits that make up these receptors must be synthesized within the cell and then transported to the plasma membrane, where they are precisely positioned in the regions adjacent to the synapse via their interactions with scaffold proteins as gephyrin, collybistin, MPP2, and radixin [53,78,79]. However, no clear evidence is available on the molecular mechanisms involved in its transport and anchoring that would support the extremely precise distribution for all GABA-A tonic subunits. On the other hand, there is evidence suggesting that diffusion, a membrane transport mechanism, could explain why imaging techniques allow the detection of all tonic receptors, both fast-acting and slow-acting, in all three synaptic regions. It has been observed that receptors embedded in the plasma membrane can move by diffusion from synaptic regions to peri- and extrasynaptic areas, and vice versa; however, this does not imply or suggest a fixed subcellular distribution [13,80].

### 2.2. Are GABA-A Tonics Suitable for the Development of Targeted Therapies?

Despite the biological importance of GABAergic tonic inhibition and it being postulated as a pharmacological target for the regular states of overexcitation through the activation of a persistent inhibitory resource, there are only a few reviews that address this topic [4,11,56,81,82,83,84,85]. Notably, there are no reports showing that these clinically important inhibitory resources are also frequently relocated in response to the functional and neurochemical environment in which they are located, highlighting their functional and pharmacological importance.

Tonic conductance plays a strategic role in the efficient detection of low local GABA levels and the subsequent restoration of the balance between excitation and inhibition at extrasynaptic and/or perisynaptic locations, and this is a long-term fine-tuning mechanism [65,86,87]. The biophysical properties of GABA-A-mediated tonic currents, such as their long duration and high affinity for the agonist, have linked them to cellular protective resources against excitotoxic injury and cell death through persistent inhibition in the presence of excessive excitation [11,65,86,87]. There are more questions than answers regarding the importance of tonic inhibition for the development of targeted therapies. In applied research, tonic inhibition is considered an alternative pharmacological target for the treatment of pathological imbalances between excitation and inhibition, such as those that induce epilepsy, Down syndrome, affective disorders, alcoholism, schizophrenia, autism, stroke, insomnia, postpartum depression, Parkinson’s disease, visual, sleep, learning, and memory disorders, and Huntington’s disease [17,82,84,85,87,88,89,90].

Although tonic subunits can be adjusted to maintain normal function using pharmacological molecules, it is worth considering that some findings indicate an alteration in neuronal function because of a gain or loss of inhibitory tonic function, which, rather than generating improvement, could lead to a deterioration of the nervous system [56].

### 2.3. Some Alterations in the Expression of GABA-A Tonic Subunits

It is not a recent discovery that changes in neurotransmitter pathways occur under pathological conditions; fluctuations in the tonic subunits α5, β3, δ, ρ2, and ρ3 have been described in a mouse YAC128 model, an animal model of Huntington’s disease (HD); the qRT-PCR analysis showed a clear increase in RNA encoding the α5 and β3 subunits as well as the ρ2 and ρ3 subunits compared to their respective controls. However, in the control group, the α5 and β3 subunits showed a decrease in their expression at the RNA level with increasing age, while the ρ2 and ρ3 subunits showed an increase in their expression with the onset of aging. Interestingly, although the expression of the δ subunit at a later age did not exceed the expression observed at an earlier age, after a decrease in its expression past this age, its expression ended up increasing in old mice in both the YAC128 group and the control groups [17]. These data suggest that tonic subunit expression becomes more prominent in old age, both in the presence and absence of Huntington’s disease. This finding is supported by studies showing that youth and pathological conditions such as epilepsy and Alzheimer’s disease are factors that can influence tonic subunit expression, such as that of GABA-A α5, β3, δ, and ρ1 [66,91,92].

### 2.4. Relocation or Overexpression and Redistribution of Tonic GABA-A Receptors? The Study Has Had Limitations

The controversy surrounding the subcellular localization of tonic subunits is considerable. Transmission electron microscopy, combined with immunogold assays, shows a recurrent redistribution of tonic subunits within the synaptic cleft, for example, when HD progresses; i.e., the usual peri- and extrasynaptic localization of each tonic subunit analyzed showed a similar population within the synaptic cleft when compared with the peri- and extrasynaptic distributions. This suggests the homogeneous localization of tonic subunits at the synaptic, perisynaptic, and extrasynaptic levels induced by the disease itself. These results are consistent with electrophysiological experiments indicating that this relocation alters the electrophysiological properties of neuronal connectivity [17]. In parallel, this immunogold analysis also indicated that, in aged control animals, the same synaptic redistribution was barely detectable but consistent.

Taken together, these latest findings suggests that the relocation of the tonic subunits is differential and appears to respond specifically to the cellular environment, so it does not appear to be a fortuitous change resulting from an alteration in the cytoskeleton or in the cytoplasm of diseased cells, which could in turn be related to a failure in the cargo and anchoring proteins responsible for the transit and localization of the tonic subunits. The redistribution of tonic resources as an apparent adaptive capacity to relocate to a hyperexcitable environment to cope with sudden cellular stress could be due to the imbalance between excitation and inhibition that sometimes characterizes aging and distinctively characterizes HD.

Despite this controversial relocation and functional involvement of tonic inhibition in regions of phasic activity in HD, there is clear evidence supporting its location within the active zone of the synapse, mainly those pointing to the α5 subunit of the GABA-A family. A key study was also performed that firmly demonstrated the synaptic adaptability of this subunit using the immunogold analysis. With the support of this subcellular approach’s experimental tool, it was determined that the α5 subunit can be distributed at both the presynaptic and postsynaptic levels, in addition to being located at the three levels of neuronal communication: (a) synaptic, (b) perisynaptic, and (c) extrasynaptic. This finding was reported in the cortex and hippocampus of rats between 35 and 70 days old and laid the groundwork for the possibility that the α5 subunit could play a functional role in both phasic and tonic distributions [93].

In support of this hypothesis, there are a few studies confirming that the GABA α5 subunit is a functional tonic resource that can also be in the classical regions where the phasic subunits are distributed. First, optogenetic stimulation findings initially indicate that this subunit contributes to the synaptic to postsynaptic currents of hippocampal CA1 interneurons containing somatostatin and nitric oxide synthase in five- to ten-week-old mice [94]. Second, under the same optogenetic experimental scheme, it was shown that, in the hippocampus of 2-week-old to 6-month-old mice, the α5 subunit is redistributed from the extrasynaptic to the synaptic regions, prolonging the inhibition of recurrent excitatory glutamatergic long-term potentiation (LTP) events [95]. LTP is a neuronal plasticity involving positive feedback; recurrent LTP involves recurrent cycles of excitatory activity, and this hyperexcitability can be generated, leading to excitotoxicity and therefore cell injury or cell death.

Furthermore, the in vitro studies of synaptic and extrasynaptic membrane fractionation of primary cultures of cortical neurons of postnatal origin (0- to 1-day-old) have also reported that the tonic α4 subunit can be located in both synaptic and extrasynaptic regions [96].

This is evidenced in several key studies addressing GABA extrasynaptic/perisynaptic localization and synaptic relocalization. Table 1 summarizes the experimental approaches, animal models, and subunits from the research presented in this section, providing a concise overview of GABA tonic relocalization.

Considering these consistent findings, it can be confirmed that the relocalization of tonic GABA-A subunits within regions of the synaptic cleft is a cellular resource that appears to be triggered by age and when there is an imbalanced predominance of excitation over inhibition in neuronal alterations. This phenomenon, observed at the microscopic level, shows us a redistribution that responds dynamically to the extracellular environment or is simply an oversynthesis of receptors specialized in tonic inhibition that are required to achieve equilibrium states when excitation has overcome inhibition.

**Figure 4:**
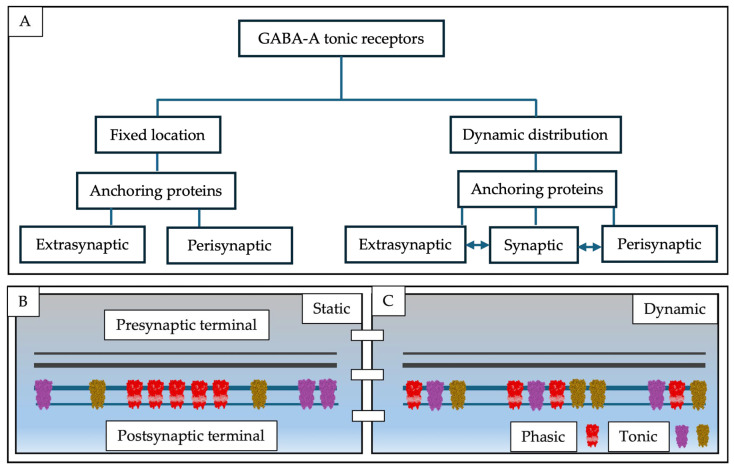
(**A**) Conceptual diagram of GABA-A tonic static location vs. dynamic distribution. (**B**) The classic peri- (golden pentamer) and extrasynaptic (magenta pentamer) distributions of tonic GABA-A subunits are represented in the diagram vs. location of synaptic pentamers (red pentamers). They are assumed to be molecular entities that are specifically located in the membrane. In pathological conditions and age-related events, it has been reported that they may be in synaptic regions to tackle cellular overexcitation more quickly and effectively. (**C**) The diagram shows that their distribution is dynamic and that they can be present in all three synaptic regions. Using in vitro methods, we could observe them especially in perisynaptic and extrasynaptic regions, suggesting rapid mobilization from the synaptic region to the other regions. It is likely that, in conditions associated with age or disease, greater inhibitory tonic expression is required to maintain balance in the face of an overexcitation event, thus homogenizing distribution across the membrane, which is only possible to observe using in vivo microscopy techniques.

Despite all this evidence, there is a school of thought supported by nanoscopy studies that show molecules in real time, which suggests that all receptors exhibit rapid and constant movement in neuronal membranes, responding to both the extracellular and intracellular environments [14]. In other words, before the era of super-resolution microscopy, were we likely observing an emergent overexpression in response to an excitatory insult that positions a greater number of tonic receptors at the synaptic level? However, they are normally found in the synaptic cleft in a low proportion and in a position dependent on time and stimulus. Therefore, is overexpression the reason why these receptors are more easily observed at different synaptic levels? At other times, did we micrograph a dynamic event and interpret it as static due to the absence of super-resolution microscopy? Like all other membrane receptors, are tonic receptors at the synaptic level involved in a process of membrane dynamics that mobilizes them between different synaptic regions for anchoring, recycling, or degradation? Are we interpreting their function based on the position in which we capture them under the transmission electron microscope and in functional studies? This controversy is summarized in Figure 4.

## 3. Future Directions

### What Can We Expect from the Dynamic Synaptic Allocation of Tonic GABA-A Resources in the Future?

Whether static or dynamic, the distribution of tonic GABA-A subunits within the regions of classical phasic subunit localization is a functionally important cellular resource and is increasingly recognized as a mechanism of cellular plasticity in the face of physiological or pathological imbalances that can be alleviated by highly GABA-sensitive resources with persistent inhibitory activity. This adaptation of synaptic overexpression or relocation that may underlie prior cellular plasticity is appropriately described with the term metaplasticity, which means plasticity of plasticity, i.e., it involves a second adaptive event that helps counteract the functional imbalances generated by a first adaptive activity, as observed in long-term potentiation [95].

To date, everything points to the clear mobilization of tonic GABA-A subunits within the active zone of neuronal communication, and this appears to be an emerging need given the need for a persistent inhibitory function to confront excitotoxic cellular insults with the aim of safeguarding cellular homeostasis. However, multiple investigations are still needed to achieve the following aims: (a) to determine the molecular identity of relocated synaptic tonic receptors; (b) to assess whether is synaptic and peri- and extrasynaptic molecular identity is differential; (c) to gauge whether it is sufficient to determine a dynamic or static event using the real-time imaging of a single molecule; (d) to describe the identity of the cargo proteins, membrane-anchoring proteins, and intracellular mechanisms responsible for positioning this type of GABAergic receptors and others receptor types, (e) to determine whether both static and dynamic locations are physiological states and the several factors they depend on; (f) to determine whether the redistribution of peri- and extrasynaptic regions in synaptic regions or overexpression is a short-term process and therefore reversible until the cellular homeostasis system is restored and long-term resources are generated to respond adequately to the GABAergic environment in the extracellular space, etc., and, (g) to assess the fate of phasic subunits when tonic resources are relocated or overexpressed.

For several years, along with the application of TEM, techniques such as fluorescence recovery after photobleaching, as well as total internal reflection fluorescence microscopy (TIRFM), have been applied to the study of the dynamics of membrane molecules, including the receptors embedded in it [97]; however, the limited resolution achieved by these techniques has not been a resource that responds adequately. At this point, super-resolution microscopy such as Photo-Activated Localization Microscopy (PALM), a variant of single-molecule localization microscopy (also known as SMLM), seems to be a tool for studying live-cell imaging and membrane dynamics; this technique achieve 10–25 nm of resolution, and, in particular, it can increase temporal resolution when connected to an ultra-fast camera for live event recording [98].

Super-resolution microscopy is a powerful tool that can provide reliable answers to the question of whether tonic receptors have a static or dynamic location. At the cellular level, this information is essential for understanding how neurons modulate, in real time, synaptic transmission at the tonic inhibition level, and the timing and intensity with which they generate signals in response to the GABAergic environment and its modulators. Furthermore, it will be possible to understand its role in synaptic depression (overinhibition) and its adaptation to states of hyperexcitability. All of this is directly related to neuronal physiology, which directly influences sensory and cognitive functions. Furthermore, pharmacologically, super-resolution microscopy can enable drug sensitivity assessments and, therefore, determine the appropriate concentrations of agonists or antagonists of these receptors required to stimulate their participation and even their expression, as well as to inhibit one or both factors. In this sense, knowing the availability, dynamics, and population of receptors is a fundamental issue for pharmacological studies associated with the treatment of anxiety, epilepsy, alcoholism, neurodegenerative diseases, attention deficit disorder, autism spectrum, etc.

## 4. Conclusions

Taken together, the reviewed evidence demonstrates that tonic GABA-A subunits may exhibit a more flexible and context-dependent subcellular distribution than previously assumed. In the static context of their recurrent relocalization from perisynaptic and extrasynaptic domains to the synaptic cleft—particularly evident during aging and under hyperexcitability—they appear to represent an adaptive cellular mechanism, and not a random consequence of cytoskeleton disruption. The findings from immunogold, electron microscopy, optogenetics, and membrane fractionation studies support the idea that tonic subunits can occupy synaptic, perisynaptic, and extrasynaptic regions, thus participating in traditional regions where the involvement of phasic receptors has been suggested, in addition to tonic receptors, which would depend on the functional demands of the neuronal environment. The previous observations made with electron micrographs, which have provided snapshots of dynamic traffic and have now been elucidated using super-resolution nanoscopy, underline the importance of receptor mobility as a fundamental element of inhibitory plasticity and metaplasticity. Despite the great advances made in super-resolution microscopy for assessing whether tonic GABA-A receptors operate in static microdomains or experience continuous trafficking through synaptic regions, crucial questions will still remain about the molecular identity of the involved pentamers, the mobility states, and the regulatory proteins. Answering these questions is essential for understanding how neurons adjust the inhibitory tone under physiological and pathological conditions, which will have direct implications for pharmacological strategies targeting GABAergic receptors in central nervous system disorders.

## Figures and Tables

**Table 1:** This table compiles the subunits examined, experimental approaches, and the corresponding models used. Both physiological conditions across developmental stages and a pathological contexts are included. Together, these studies provide comparative evidence regarding the localization patterns of α5, α4, β3, δ, ρ2, and ρ3 subunits.

Subunit(s)	Method	Experimental Model	Disease or Age	Ref.
α5, β3, δ, ρ2, ρ3	Immunogold	mouse	Huntington’s disease model	[17]
α5	Immunogold	rat	Between 35 and 70 days old	[93]
α5	Optogenetic	mouse	5 to 10-week-old	[94]
α5	Optogenetic	mouse	2 weeks to 6 months of age	[95]
α4	Membrane fractionation	rat primary cortical cell culture neuron	18 days in vitro	[96]

## Data Availability

No new data were created or analyzed in this study. Data sharing is not applicable to this article.

## Abbreviations

ATD	Amino terminal domain
Ax	Axon
CTD	Carboxi terminal domain
Den	dendrite
EC-50	half-maximal effective concentration
GABA	γ-Aminobutyric acid
GAD	glutamate decarboxylase
gc	glial cell
HD	Huntington disease
mt	mitochondria
mvb	multivesicular bodies
NC-IUPHAR	Nomenclature and Standards Committee of the International Union of Basic and Clinical Pharmacology
nf	neurofilaments
PALM	Photo-Activated Localization Microscopy
PrSm	presynaptic membrane
PrS	presynaptic terminals
PtSm	postsynaptic membrane
SCl	synaptic cleft
SMLM	single-molecule localization microscopy
sv	synaptic vesicles
TM (1-4)	Transmembrane domain
